# Medical students‘ leadership competence in health care: development of a self-assessment scale

**DOI:** 10.1186/s12909-024-06037-2

**Published:** 2024-11-06

**Authors:** Barbara Ogurek, Sigrid Harendza

**Affiliations:** 1 Academy for Training and Career, University Medical Centre Hamburg-Eppendorf, Hamburg, Germany; 2 III. Department of Internal Medicine, University Medical Centre Hamburg-Eppendorf, Hamburg, Germany

**Keywords:** Competences, Leadership, Medical Leadership Competence Framework (MLCF), Medical students, Professional identity, Self-assessment, Undergraduate medical education

## Abstract

**Background:**

Medical leadership plays an increasing role already in early career stages. Undergraduate medical students in the transition to postgraduate education feel not well prepared for their leadership roles. While leadership curricula have been developed, instruments for students’ self-assessment of leadership competences as part of their professional development are still missing. The aim of our study was to develop a self-assessment scale for undergraduate medical students’ leadership competences.

**Methods:**

The medical leadership competence scale (MeLeCoS) for undergraduate medical students was developed in twelve steps. For item generation, we employed the Medical Leadership Competence Framework (MLCF), which is also used as a framework for many leadership curricula and includes five leadership domains for three fields of education: undergraduate education, postgraduate education, and continuing practice. In a pretest, 67 items were tested with *n* = 88 undergraduate medical students. For content validation we performed group discussions with a total of 17 students. After item reduction a test-version with 45 items and a 5-point Likert scale (1: ‘never’, 2: ‘rarely’, 3: ‘sometimes’, 4: ‘often’, 5: ‘always’) was used in a test-sample of final-year students (*n* = 129). Descriptive statistics and factor analyses were performed.

**Results:**

The final version of the MeLeCoS includes 37 items and the scale’s Cronbach’s alpha was 0.87. Six factors could be identified and two of them, respectively, represent leadership aspects from the following three areas: (1) the medical students themselves, (2) the interrelation of the medical students with a healthcare organisation regarding general management and improvement, and (3) general leadership aspects of medical students within undergraduate medical studies and the healthcare system. The overall mean of the MeLeCoS was 3.50 ± 0.39. Factor 2 (‘Demonstrating responsible behaviour and shaping relations’) reached the highest mean (4,36 ± 0.37) and factor 5 (‘Promoting improvement and innovation in undergraduate medical education’ the lowest (1.91 ± 0.87).

**Conclusions:**

The medical leadership competence self-assessment scale (MeLeCoS) is a reliable instrument for undergraduate medical students’ self-assessment of leadership competence with good content validity. It could be used for students’ self-reflection on leadership competences in addition to rater-based assessments in leadership curricula and for longitudinal development of students’ professional identity.

**Supplementary Information:**

The online version contains supplementary material available at 10.1186/s12909-024-06037-2.

## Background

In the growing complexity of the healthcare environment physicians’ leadership plays an increasing role in clinical, administrative, and management decisions [1–3]. Already in early career stages after graduation, physicians have a high level of responsibility in the formal or informal leadership role within healthcare teams [4, 5]. At later career stages, it is expected that they recognize the need for change as well as drive this change and improve the quality of patient care as part of their leadership role [6, 7]. However, physicians reported that they felt not prepared for such leadership tasks within the healthcare system [8, 9]. To address this lack of leadership competences, leadership trainings were recommended and established in residency training [10, 11]. To bridge the gap between undergraduate and postgraduate training with respect to the leadership role and to improve the transition process, leadership trainings are increasingly established in undergraduate education [12–14].

With respect to teaching content for the development of a leadership curriculum within undergraduate medical education, students identified relevant leadership topics, e.g. dealing with conflicts, time management or investment principles, where they did not feel well prepared [15]. Furthermore, different aspects of formal and informal leadership have been suggested to be included in leadership curricula [16]. An analysis of 24 leadership curricula in undergraduate medical training revealed that most of them were longitudinally designed over periods up to four years [17]. More than 75% of the curricula analyzed in this study addressed at least three of the five leadership domains of the Medical Leadership Competence Framework (MLCF) [18]. In an analysis of 25 undergraduate leadership curricula at US medical schools most leadership themes that were identified could be assigned to the two MLCF domains ‘Demonstrating personal qualities’ and ‘Working with others’ [19]. Didactic approaches in such curricula show a great variety depending on the leadership skill on which they focus and include seminars, lectures, workshops, or simulation [20–23].

Assessing the impact of leadership curricula on students’ leadership competence is difficult and not always implemented [17]. In an analysis of leadership curricula in Australian and New Zealand medical schools, 10 of 17 assessed leadership competences in their undergraduate medical students, e.g., by written examinations or practical exams like Mini-CEX [24]. Additionally, self-assessment and reflection are important assets in the training and development of professional behaviour [25] such as leadership. Furthermore, final-year medical students would appreciate feedback and self-assessment questionnaires as an important approach to measure and improve their leadership competence [26]. While self-assessment instruments for medical leadership competences are available for physicians [27, 28], no such instruments are available for undergraduate students even though the MLCF covers learning objectives for undergraduate students’ medical leadership competences [18]. Therefore, the aim of our study was to develop a self-assessment questionnaire for medical leadership based on the five MLCF dimensions for undergraduate medical students to be used for students’ individual development of leadership competence.

## Methods

### Construction of the medical students’ leadership competence self-assessment scale (MeLeCoS)

**Fig. 1:**
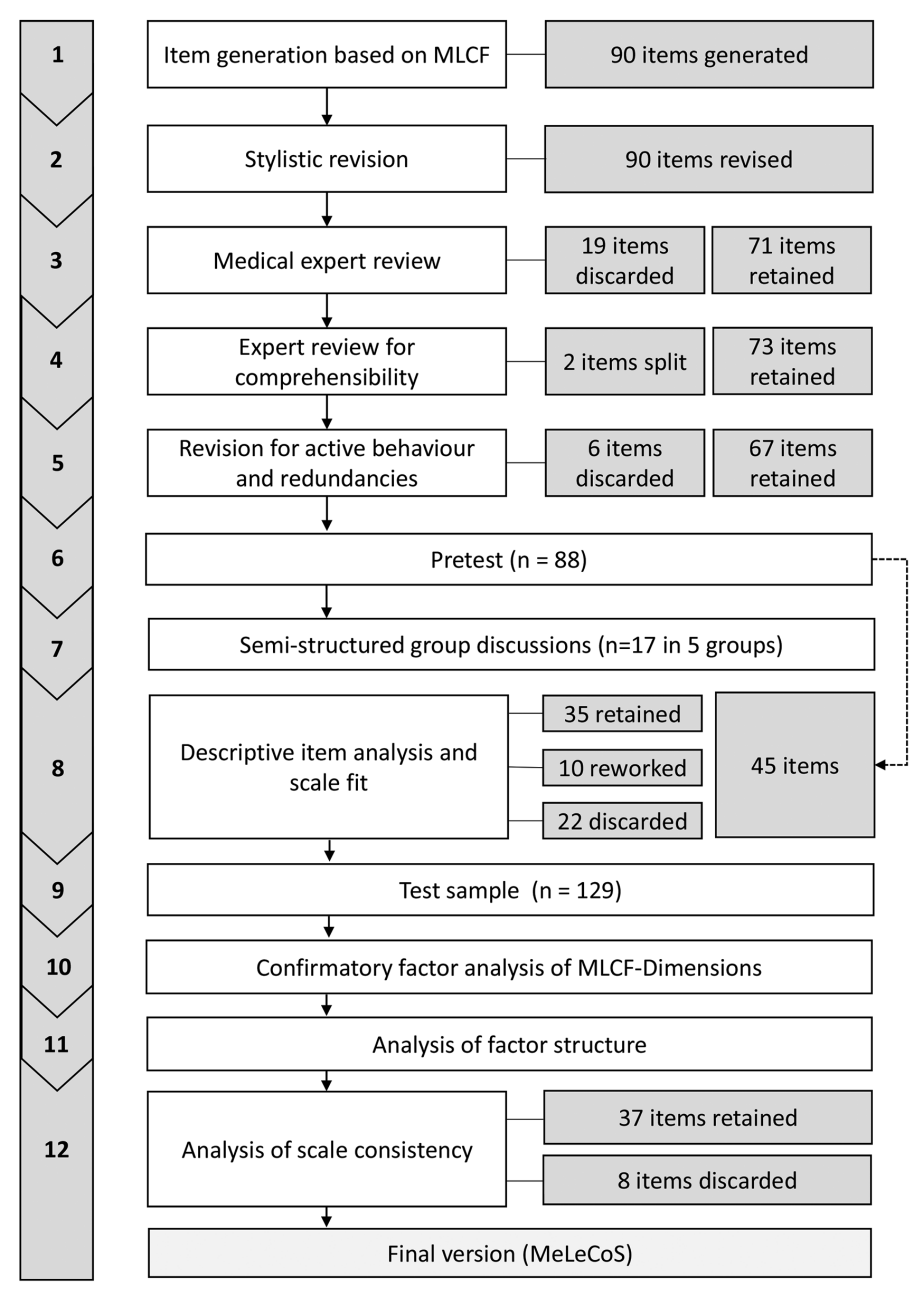
Development steps of the medical students’ leadership competence self-assessment scale MeLeCoS

The self-assessment scale for medical leadership competence was developed in twelve steps (Fig. 1). The items for a medical leadership competence self-assessment scale for undergraduate medical students were based on the Medical Leadership Competency Framework (MLCF) [18]. This framework consists of the five domains ‘Demonstrating personal qualities’, ‘Working with others’, ‘Managing services’, ‘Improving services’, and ‘Setting directions’ with examples for undergraduate and postgraduate training as well as continuing practice. In step 1 of constructing the instrument, items were generated from undergraduate and postgraduate examples of the framework’s five domains following the framework and supplemented with additional items matching with the requirements of the six-year undergraduate medical education in Germany. Of the 90 items in total, 88 were based on the MLCF and two were generated based on German requirements to adapt aspects of the domain ‘Setting directions’ to the situation in Germany. The items were formulated as active behaviour by BO, an educationalist. In step 2, the items were stylistically revised. SH, a physician (internist) and medical education expert reviewed the items for their medical content and 19 items were removed (step 3). The items were discussed with two sociologists and one psychologist from the research team to improve wording and comprehensibility of the items (step 4). Eventually, 2 items were split and some were stylistically reworked resulting in 73 items (step 4). These items were revised with respect to exclusively representing active behaviour in the final wording and critically reviewed for redundancies (step 5). Six items were excluded in this step. Following the theoretical framework of the MLCF [18] its five domains were maintained in all steps of item reduction (‘Demonstrating personal qualities’: 17 items, ‘Working with others’: 14 items, ‘Managing services’: 12 items, ‘Improving services’ 13 items, and ‘Setting directions’: 11 items).

For pretesting, the resulting 67 items were implemented in Lime Survey Cloud version 6.3.9 (LimeSurvey GmbH, Hamburg, Germany) as a digital questionnaire (step 6). To facilitate responses, the items were categorized in three fields of specific leadership behaviour (18 general leadership items, 21 hospital-related leadership items, and 28 study-related leadership items). All items were randomized within the three categories, each category on one page, and the order of the three categories was randomized, too. The five domains of the MLCF were not disclosed to the participants to avoid answer biases with respect to the headlines of the domains. The sociodemographic data placed at the end of the questionnaire included sex, age, and advancement in the final year. The frequency of expression of the specific behaviour of the 67 items could be rated on a 5-point Likert scale (1: ‘never’, 2: ‘rarely’, 3: ‘sometimes’, 4: ‘often’, 5: ‘always’). For pretesting and to generate more information about the fitting of the items to the real experience of medical students, the additional answering option ‘not applicable” was provided. The study was performed at the Medical Faculty Hamburg which does not provide a leadership curriculum during undergraduate medical education. In May and June 2023, 422 final-year students were invited via e-mail for a pretest and 106 students started with the self-assessment of which 88 completed the survey (response rate: 25.1%, completion rate: 83.0%). To enhance content validity, five semi-structured group discussions about the general understanding of medical leadership were conducted by BO with 17 of 42 invited final-year medical students (step 7). In the discussions, which lasted between 45 and 65 minutes, the students were asked to discuss their own examples of medical leadership as well as to provide their ideas of ideal medical leadership behaviour. The discussions were double audio recorded, transcribed with f4x and reworked twice. BO evaluated the transcripts by qualitative content analysis [29] and discussed her findings with SH. All medical leadership aspects raised in the discussions were already included in the questionnaire. Hence, no further items needed to be added to the pretest-version of the questionnaire. In step 8, the results of the pretests were analysed based on the reliability of the items related to the five MLCF-dimensions using IBM SPSS Statistics 29.0.0.0 [30]. The items with very low scale fit and low or negative impact on factor reliability were rejected. Four items could be reduced by combining with other items. Three items were maintained despite medium fit due to theoretical implications confirmed in the group discussions. Due to fitting of all items to the real experiences of medical students in Germany, there was no need any more to provide the answering option ‘not applicable’ in the scale. This process resulted in a test version of the questionnaire with 45 items (‘Demonstrating personal qualities’: 11 items, ‘Working with others’: 8 items, ‘Managing services’: 11 items, ‘Improving services’: 6 items, ‘Setting directions’: 9 items). In the questionnaire, the items were categorized (17 general leadership items, 12 hospital-related leadership items, and 16 study-related leadership items) and randomized individually in the same way as in the pretest.

### Testing of the medical students’ leadership competence self-assessment instrument

The test version of the digital questionnaire was used between November 2023 and January 2024. Overall, 716 undergraduate medical students in their final year were invited via e-mail. In total, 160 students started with the self-assessment and 129 complete datasets could be included in the analyses (response rate: 22.3%, completion rate: 80.6%) (step 9). Participation was voluntary and anonymous. All participants gave their written consent. The study was performed in accordance with the Declaration of Helsinki and the Ethics Committee of the Chamber of Physicians, Hamburg. Germany, approved this study (PV3649).

### Data analysis

Data analysis was performed using IBM SPSS Statistics version 29.0.0.0 [30] and computing software R version 4.3.1 (R Core Team, Vienna, Austria). Using the lavaan package [31] a confirmatory factor analysis was run to test the theoretical factor structure of the MLCF (step 10). To analyse the internal consistency of the five dimensions assumed by the MLCF, Cronbach’s alpha was calculated using SPSS [30].

In a next step (step11), explorative factor analyses were performed for possible identification of a fitting factor structure, because the MLCF structure could not be confirmed. To determine the number of factors, parallel analyses were run using R psych package [32]. To find the best combination of items within the suggested number of factors, explorative factor analyses with principal axes analysis and oblimin rotation using the GPArotation [33] and the lavaan [31] package were performed for all options suggested by parallel analyses. The resulting three different models of item combination were then inspected. To assess internal consistency, Cronbach’s alphas were calculated using IBM SPSS and the item scale correlation was examined (step 12). For all three models, all items negatively influencing the internal consistency or with low item-scale correlation (< 0.3) [34] were removed resulting in the final version of the MeLeCoS.

## Results

The 129 final-year students had a mean age of 27.8 ± 3.8 years and included 85 (65,9%) female, 43 (33,3%) male and 1 (0.8%) diverse participants. The theoretical factor structure of the five MLCF dimensions could not be validated with confirmatory factor analysis (step 10). The parallel analyses (step 11) led to the options to assume either four, six or seven factors. The best fit could be reached with the seven-factor-model. Within the seven-factor-model, one factor covered only one item. This item, acceptable load on another factor, was regrouped and the seventh factor was dropped resulting in six factors. Excluding eight items because of negative impact on the internal consistency of the different factors and low item-scale correlation (step 12), the remaining 37 items allocated to the six factors provided a good fitting model. The final version of the MeLeCoS is shown in supplement 1.

Table 1 shows the six MeLeCoS-factors including their individual internal consistencies and the number of items per factor. Factor 1 (‘Achieving learning and reflecting on performance’) and factor 2 (‘Demonstrating responsible behaviour and shaping relations‘) include leadership aspects regarding the medical students themselves. Factor 3 (‘Fostering personal development and promoting quality improvement’) and factor 4 (‘Developing self-management and supporting management in healthcare’) reference to the interrelation of the medical students with a healthcare organisation regarding general management and improvement. Factor 5 (‘Promoting improvement and innovation in undergraduate medical education’) and factor 6 (‘Introducing systemic perspectives into organizations’) deal with aspects of leadership and management in general within undergraduate medical studies and the healthcare system. The overall Cronbach’s alpha for the MeLeCoS is 0.87. A table describing the factor structure can be found in supplement 2 and a table including the p-values and confidence intervals in supplement 3.

**Table 1:** MeLeCoS-factors and their internal consistency

Factor	Cronbach’s alpha	Number of items
(1) Achieving learning and reflecting on performance	0.75	5
(2) Demonstrating responsible behaviour and shaping relations	0.74	9
(3) Fostering personal development and promoting quality improvement	0.71	6
(4) Developing self-management and supporting management in healthcare	0.77	8
(5) Promoting improvement and innovation in undergraduate medical education	0.82	5
(6) Introducing systemic perspectives into organizations	0.65	4

The overall results of the MeLeCoS on factor and item level are shown in Table 2. The overall mean of the MeLeCoS is 3.50 ± 0.39. Factor 2 (‘Demonstrating responsible behaviour and shaping relations’) and factor 1 (‘Achieving learning and reflecting on performance’) reached the highest mean values, 4.36 ± 0.37 and 4.07 ± 0.59, respectively. The lowest mean value, 1.91 ± 0.87, was found for factor 5 (‘Promoting improvement and innovation in undergraduate medical education’). The other three factors reached means between 3.21 ± 0.64 and 3.64 ± 0.58. On item level, the two items ‘I behave ethically towards fellow students and teachers (e.g. I do not discriminate against anyone on the basis of cultural origin).’ and ‘I behave ethically towards patients in clinical situations (e.g. I treat all patients equally, regardless of their social background).’ from factor 2 (‘Demonstrating responsible behaviour and shaping relations’) attained the highest mean, 4.74 ± 0.58 and 4.74 ± 0.44, respectively. The item ‘I am involved in the student council and/or committees.’ from factor 5 (‘Promoting improvement and innovation in undergraduate medical education’) showed the lowest mean (1.67 ± 1.07) followed by the item ‘I am involved in student groups to improve the general conditions for studying (e.g. support for students with children).’ with 1.89 ± 1.13 from the same factor. The item-scale correlations for all items are provided in supplement 4.

**Table 2:** Items and factor structure of MeLeCoS

Items	Item M ± SD	Factor M ± SD	Factor No.
I question whether I have delivered the best possible performance.	4.43 ± 0.67		
I reflect on my performance at the end of each study period or semester.	3.95 ± 1.05		
I can control my self-learning well (e.g. I start studying early for exams).	4.19 ± 0.87	4.07 ± 0.59	**1**
I compare my knowledge and practices with those of my peers to question both content and actions.	3.82 ± 0.81		
I communicate goals clearly in working or learning groups so that we can work together to achieve them.	3.96 ± 0.70		
In controversial discussions, I make sure that the views of all participants are heard before decisions are made.	3.72 ± 0.89		
I take responsibility for the active role assigned to me in a team (e.g. minute taker).	4.39 ± 0.73		
I motivate others in group work.	3.71 ± 0.80		
I behave responsibly during my studies (e.g. I contribute to a good working atmosphere during group work).	4.43 ± 0.61		
I behave ethically towards fellow students and teachers (e.g. I do not discriminate against anyone on the basis of cultural origin).	4.74 ± 0.58	4.36 ± 0.37	**2**
I behave responsibly during clinical training, e.g. during a clinical clerkship.	4.71 ± 0.49		
I behave ethically towards patients in clinical situations (e.g. I treat all patients equally, regardless of their social background).	4.74 ± 0.44		
I can build a professional relationship with patients.	4.45 ± 0.54		
In history taking, I encourage patients to share their perspective.	4.34 ± 0.68		
I use information from others, e.g. feedback, to continue my learning.	4.25 ± 0.71		
If I recognize the influence of poor performance on the quality of results, then I discuss this with the people involved.	3.27 ± 0.84		
In groups, I try to discuss identified problems further.	3.79 ± 0.79		
After critical incidents, I voluntarily participate in the review of work processes in the affected work area.	3.33 ± 0.98	3.64 ± 0.58	**3**
I look for role models from whom I can learn something about the healthcare system or healthcare organizations.	3.76 ± 1.03		
I organize additional extracurricular learning opportunities for myself (e.g. study groups with fellow students).	3.43 ± 1.04		
In emotional situations, e.g. when receiving very critical feedback, I communicate in a controlled and objective manner.	3.78 ± 0.70		
I am involved in research (e.g. through my own research projects or research supporting activities).	3.24 ± 1.32		
I seek additional learning opportunities to recognize how decisions are made in the light of new knowledge and information.	3.16 ± 1.06		
I support other students in their studies (e.g. as a mentor or by providing learning materials).	3.25 ± 1.16		
I seize learning opportunities to understand the basic principles of healthcare financing.	2.60 ± 1.04	3.21 ± 0.64	**4**
During my clinical training, I contemplate the use of resources (e.g. when ordering laboratory diagnostics).	3.80 ± 0.74		
I discuss the opportunities and limitations of change projects in student groups (e.g. the introduction of digital medical records).	2.81 ± 1.15		
When changes are introduced in medical procedures (e.g. shortening the length of inpatient treatment), I keep myself informed about their effectiveness.	3.05 ± 0.93		
I am involved in the student council and/or committees.	1.67 ± 1.07		
I take part in projects or committees to improve undergraduate medical studies and teaching.	2.08 ± 1.13		
I am involved in student groups to improve the general conditions for studying (e.g. support for students with children).	1.89 ± 1.13		
I take on leadership roles in a student group to implement teaching innovations (e.g. ultrasound tutorials).	1.93 ± 1.19	1.91 ± 0.87	**5**
I am involved in student groups to implement teaching innovations (e.g. ultrasound tutorials).	1.96 ± 1.18		
I take responsibility for finances or resource planning in an organization (e.g. in a club or a group).	2.51 ± 1.35		
I am actively involved in a change project (e.g. a reorganization in a club).	2.69 ± 1.26		
I share information so that others can understand me better.	4.23 ± 0.64	3.26 ± 0.73	**6**
I am able to steer group dynamic processes (e.g. by involving quieter group participants).	3.60 ± 0.79		
**Total**	**3.50 ± 0.39**		

Factor 1: Achieving learning and reflecting on performance; Factor 2: Demonstrating responsible behaviour and shaping relations; Factor 3: Fostering personal development and promoting quality improvement; Factor 4: Developing self-management and supporting management in healthcare; Factor 5: Promoting improvement and innovation in undergraduate medical education; Factor 6: Introducing systemic perspectives into organizations

## Discussion

In a stepwise process we developed the self-assessment scale MeLeCoS for leadership competence of undergraduate medical students based on the leadership competence framework MLCF [18]. The MeLeCoS consists of 6 factors with a total of 37 items and shows a good Cronbach’s alpha of 0.87. To our surprise, the factor structure of the MeLeCoS does not follow the MLCF domains but comprises three different areas of medical leadership. Factor 1 (‘Achieving learning and reflecting on performance’) and factor 2 (‘Demonstrating responsible behaviour and shaping relations’) comprise the area of medical students’ individual leadership competence in healthcare. Factor 3 (‘Fostering personal development and promoting quality improvement’) and factor 4 (‘Developing self-management and supporting management in healthcare’) are related to the area of students’ leadership competence in relation to the health care system. Factor 5 (‘Promoting improvement and innovation in undergraduate medical education’) and factor 6 (‘Introducing systemic perspectives into organizations’) include the area of students’ leadership competence on an organizational level. These three areas we identified for undergraduate students match with the three levels of leadership competence suggested for designing a postgraduate leadership curriculum in healthcare: (1) individual and social leadership competence, (2) professional and methodological leadership competence, and (3) conceptual leadership competence [35]. Competences that can be assigned to these three areas are also part of the competences to be achieved for the leadership role in the CanMEDS framework [36] and can be found in the development and evaluation of other medical leadership competence frameworks [7, 37, 38]. In the design of leadership curricula, a specific focus is sometimes set to one of these three areas, e.g., service learning [39]. With its six factors covering the three areas and levels of leadership competence, respectively, the MeLeCoS can be used for undergraduate medical students’ self-assessment of leadership competence to accompany any leadership curriculum independently of its focus to encourage students’ self-reflection.

The participating students in our study who were not trained for specific leadership competences in their undergraduate studies rated themselves highest (> 4 on a 5-point-scale) on average for the factors 1 and 2 which are related to individual and social leadership competences in the healthcare system. This could be due to developing individual leadership competences as part of developing medical professionalism where individual competences can overlap with different roles [40–42]. For instance, within the area of individual leadership competences, two items from factor 2 addressing ethical behaviour towards fellow students and teachers and towards patients in clinical situations, respectively, received the highest mean values (4.74). Ethical behaviour has been suggested to be included as a professional competence in undergraduate curricula [43] but also resembles a leadership competence from the domain ‘Demonstrating personal qualities’ of the MLCF [18], which includes specific competences longitudinally from undergraduate via postgraduate training to the continuing practice field. Students in our study rated themselves lowest (< 2 on a 5-point-scale) for factor 5, which includes items from the area of leadership competence on an organizational level in the MeLeCoS resembling support or development of the undergraduate medical curriculum from the MLCF dimension ‘Supporting services’ [18]. Self-assessment values of factor 5 would probably have been higher if our undergraduate students had been more involved in educational activities like teaching rotations during their undergraduate training [44], creating and teaching courses as peer-teachers [45], or working as student representatives on curriculum committees [46].

Self-reflection and taking responsibility are two very important aspects of professional development in undergraduate and postgraduate medical education [47]. Hence, the MeLeCoS’ application as a self-reflection tool, e.g., as part of multisource feedback [48], would be most useful in more than 25% of undergraduate leadership curricula that have been found to include all five MLCF dimensions [17] and, therefore, teaching aspects from all three areas of leadership competence. For the more than 75% of the leadership curricula that addressed at least three of the five MLCF domains [17], items from the different MeLeCoS factors being related to the respective MLCF could be used for medical students’ self-assessment and reflection, which are important for personal development [49]. Based on the longitudinal curricular design of many medical leadership curricula in undergraduate education [17], the MeLeCoS could be used to measure the progress of medical students’ leadership competence over a period of time in a self-reflective process. For instance, self-assessment with the MeLeCoS could take place both in the beginning and in advanced stages of a leadership curriculum to support students’ reflection of their personal development. Combined with assessments after the leadership courses it could provide important information about the transfer from learning into personal behaviour, which resembles the second highest level of learning [50].

Our study has several limitations. One limiting factor is that the medical content of the items was only reviewed by one physician (SH) with clinical and educational expertise. Another limitation is that all students participated voluntarily, which could have let to the bias of selecting very motivated students or students that are particularly interested in leadership. Furthermore, the sample size was relatively small for performing confirmatory factor analysis which increased the risk for model overfitting and requires further studies with an independent sample of a larger number of participants. Also, performing CFA before reliability analysis might have been advisable to first consolidat subscales and secondly optimizing their reliability. Even though the MeLeCoS had a Cronbach’s alpha of 0.87, factor 6 (‘Introducing systemic perspectives into organizations’) only reached a just below acceptable Cronbach’s alpha of 0.65, presumably due to the low number of four items compared to five to nine items for the other factors. Another limiting aspect of this study is, that several items of factor 2, ‘Demonstrating responsible behaviour and shaping relations’, have a right-skewed distribution. Such an effect, especially with items related to ethics, for instance, that drive socially desirable answers could be observed, e.g., with another scale used for self-assessment of professional identity [51]. Nevertheless, these items are needed to complete the leadership understanding and to include all aspects of leadership based on the MLCF [18]. When working with the MeLeCoS, students will need some training on how to provide honest self-assessment to benefit from the following reflection [52]. Applying the MLCF as basis for designing the MeLeCoS is a strength of our study, because the MLCF is frequently used to design medical leadership curricula [17, 19]. Another strength is using focus groups with final-year medical students to check and improve face validity of the MeLeCoS in the process of item generation. Using the MeLeCoS in a digital version with randomised item order reduced possible negative influences in answering behaviour induced by a fixed item order. When used in a non-European context, it could be necessary that the answering option N/A needs to be added to some items due to different educational situations, e.g., participation in student committees, to prevent false left-skewed distribution especially with regard to items in factor 5. With its three areas and six factors the MeLeCoS covers a comprehensive range of undergraduate medical students’ formal and informal leadership roles [16] and can be used in undergraduate medical education. Since the MLCF covers examples of leadership behaviour on three career stages, ‘undergraduate’, ‘postgraduate’ and ‘continuing practice’ [18], further self-assessment tools could be developed based on the MeLeCoS with items adapted to the respective career level.

## Conclusions

The medical leadership self-assessment scale (MeLeCoS) is a reliable instrument for undergraduate medical students’ self-assessment of their leadership competence with good content validity based on the Medical Leadership Competence Framework (MLCF). With its items covering operationalized aspects of formal and informal leadership roles on an undergraduate level it could be used for students’ self-reflection on leadership competences in addition to rater-based assessment in leadership curricula. It could also be employed longitudinally in undergraduate education for self-reflections of leadership competence as part of professional identity formation. Since the MLCF also includes learning objectives for leadership competences in postgraduate education and continuing practice, the MeLeCoS could serve as template for designing self-assessment instruments of leadership competences for these medical training phases.

## Electronic supplementary material

Below is the link to the electronic supplementary material.

Supplement 1: Medical students’ leadership competence self-assessment scale (MeLeCoS)

Supplement 2: MeLeCoS factor structure

Supplement 3: MeLeCoS p-values and confidence intervals

Supplement 4: MeLeCoS item scale correlations

## Data Availability

All data and materials are available from the manuscript.

## Abbreviations

M: Mean
MeLeCoS: Medical Leadership Competence Scale
Mini-CEX: Mini Clinical Evaluation Exercise
MLCF: Medical Leadership Competency Framework
NHS: National Healthcare Service
SD: Standard deviation
