# Supplementary material for: Medical students‘ leadership competence in health care: development of a self-assessment scale
Source: BMC Med Educ. 2024 Nov 6;24:1275. doi: 10.1186/s12909-024-06037-2 (PMC11542227; doi:10.1186/s12909-024-06037-2)
Supplement: Supplementary file 1 — Supplement 1: Medical students’ leadership competence self-assessment scale (MeLeCoS) [file 12909_2024_6037_MOESM1_ESM.docx]

**Supplement 1: Medical students’ leadership competence self-assessment scale (MeLeCoS)**

| **Item** | **Dimension MLCF** |
| --- | --- |
| **General leadership behaviour** |  |
| I use information from others, e.g. feedback, to continue my learning. | Demonstrating personal qualities |
| In emotional situations, e.g. when receiving very critical feedback, I communicate in a controlled and objective manner. | Demonstrating personal qualities |
| In controversial discussions, I make sure that the views of all participants are heard before decisions are made. | Working with others |
| I am involved in research (e.g. through my own research projects or research supporting activities). | Managing services |
| I take responsibility for finances or resource planning in an organization (e.g. in a club or a group). | Managing services |
| I take responsibility for the active role assigned to me in a team (e.g. minute taker). | Managing services |
| I motivate others in group work. | Managing services |
| If I recognize the influence of poor performance on the quality of results, then I discuss this with the people involved. | Managing services |
| I question whether I have delivered the best possible performance. | Managing services |
| After critical incidents, I voluntarily participate in the review of work processes in the affected work area. | Managing services |
| I am actively involved in a change project (e.g. a reorganization in a club). | Improving services |
| In groups, I try to discuss identified problems further. | Setting directions |
| I seek additional learning opportunities to recognize how decisions are made in the light of new knowledge and information. | Setting directions |
| I share information so that others can understand me better. | Setting directions |
| I look for role models from whom I can learn something about the healthcare system or healthcare organizations. | Setting directions |
| I am able to steer group dynamic processes (e.g. by involving quieter group participants). | Setting directions |
| **Leadership behaviour during medical studies** |  |
| I reflect on my performance at the end of each study period or semester. | Demonstrating personal qualities |
| I can control my self-learning well (e.g. I start studying early for exams). | Demonstrating personal qualities |
| I compare my knowledge and practices with those of my peers to question both content and actions. | Demonstrating personal qualities |
| I organize additional extracurricular learning opportunities for myself (e.g. study groups with fellow students). | Demonstrating personal qualities |
| I behave responsibly during my studies (e.g. I contribute to a good working atmosphere during group work). | Demonstrating personal qualities |
| I behave ethically towards fellow students and teachers (e.g. I do not discriminate against anyone on the basis of cultural origin). | Demonstrating personal qualities |
| I am involved in the student council and/or committees. | Working with others |
| I support other students in their studies (e.g. as a mentor or by providing learning materials). | Working with others |
| I seize learning opportunities to understand the basic principles of healthcare financing. | Managing services |
| I take part in projects or committees to improve undergraduate medical studies and teaching. | Improving services |
| I am involved in student groups to improve the general conditions for studying (e.g. support for students with children). | Improving services |
| I take on leadership roles in a student group to implement teaching innovations (e.g. ultrasound tutorials). | Improving services |
| I am involved in student groups to implement teaching innovations (e.g. ultrasound tutorials). | Improving services |
| I communicate goals clearly in working or learning groups so that we can work together to achieve them. | Setting directions |
| **Leadership behaviour during clinical education** |  |
| I behave responsibly during clinical training, e.g. during a clinical clerkship. | Demonstrating personal qualities |
| I behave ethically towards patients in clinical situations (e.g. I treat all patients equally, regardless of their social background). | Demonstrating personal qualities |
| I can build a professional relationship with patients. | Working with others |
| In history taking, I encourage patients to share their perspective. | Working with others |
| During my clinical training, I contemplate the use of resources (e.g. when ordering laboratory diagnostics). | Managing services |
| I discuss the opportunities and limitations of change projects in student groups (e.g. the introduction of digital medical records). | Improving services |
| When changes are introduced in medical procedures (e.g. shortening the length of inpatient treatment), I keep myself informed about their effectiveness. | Setting directions |
