# Supplementary material for: Medical students‘ leadership competence in health care: development of a self-assessment scale
Source: BMC Med Educ. 2024 Nov 6;24:1275. doi: 10.1186/s12909-024-06037-2 (PMC11542227; doi:10.1186/s12909-024-06037-2)
Supplement: Supplementary file 2 — Supplement 2: MeLeCoS factor structure [file 12909_2024_6037_MOESM2_ESM.docx]

**Supplement 2**: MeLeCoS factor structure

| **Items** | **Factor (1)** | **Factor (2)** | **Factor (3)** | **Factor (4)** | **Factor (5)** | **Factor (6)** |
| --- | --- | --- | --- | --- | --- | --- |
| I question whether I have delivered the best possible performance. | 0.545 | -0.034 | -0.004 | 0.019 | -0.009 | -0.015 |
| I reflect on my performance at the end of each study period or semester. | 0.463 | 0.031 | 0.128 | 0.308 | 0.012 | -0.085 |
| I can control my self-learning well (e.g. I start studying early for exams). | 0.486 | 0.039 | 0.040 | 0.058 | -0.050 | -0.037 |
| I compare my knowledge and practices with those of my peers to question both content and actions. | 0.424 | 0.004 | 0.095 | 0.047 | 0.009 | 0.005 |
| I communicate goals clearly in working or learning groups so that we can work together to achieve them. | 0.337 | 0.019 | -0.047 | 0.046 | 0.007 | 0.142 |
| In controversial discussions, I make sure that the views of all participants are heard before decisions are made. | 0.054 | 0.326 | -0.087 | 0.143 | -0.121 | 0.362 |
| I take responsibility for the active role assigned to me in a team (e.g. minute taker). | 0.166 | 0.160 | 0.071 | -0.026 | 0.044 | 0.029 |
| I motivate others in group work. | 0.238 | 0.187 | 0.033 | 0.064 | -0.048 | 0.201 |
| I behave responsibly during my studies (e.g. I contribute to a good working atmosphere during group work). | 0.115 | 0.271 | -0.099 | -0.049 | -0.086 | 0.057 |
| I behave ethically towards fellow students and teachers (e.g. I do not discriminate against anyone on the basis of cultural origin). | -0.163 | 0.334 | -0.051 | 0.064 | -0.036 | 0.027 |
| I behave responsibly during clinical training, e.g. during a clinical clerkship. | 0.031 | 0.213 | 0.130 | -0.060 | -0.049 | 0.007 |
| I behave ethically towards patients in clinical situations (e.g. I treat all patients equally, regardless of their social background). | -0.038 | 0.280 | -0.020 | 0.028 | 0.050 | -0.024 |
| I can build a professional relationship with patients. | 0.089 | 0.272 | -0.041 | 0.120 | 0.095 | -0.019 |
| In history taking, I encourage patients to share their perspective. | 0.120 | 0.348 | 0.113 | -0.026 | 0.067 | -0.104 |
| I use information from others, e.g. feedback, to continue my learning. | 0.144 | 0.224 | 0.228 | -0.136 | 0.028 | 0.142 |
| If I recognize the influence of poor performance on the quality of results, then I discuss this with the people involved. | 0.010 | -0.059 | 0.728 | 0.064 | -0.043 | 0.006 |
| In groups, I try to discuss identified problems further. | 0.217 | 0.209 | 0.206 | -0.054 | 0.020 | 0.131 |
| After critical incidents, I voluntarily participate in the review of work processes in the affected work area. | -0.029 | 0.110 | 0.464 | 0.128 | -0.021 | 0.113 |
| I look for role models from whom I can learn something about the healthcare system or healthcare organizations. | 0.164 | 0.038 | 0.231 | 0.105 | 0.115 | 0.148 |
| I organize additional extracurricular learning opportunities for myself (e.g. study groups with fellow students). | 0.183 | 0.131 | 0.256 | 0.046 | 0.095 | 0.021 |
| In emotional situations, e.g. when receiving very critical feedback, I communicate in a controlled and objective manner. | -0.095 | 0.041 | 0.138 | 0.298 | 0.075 | -0.041 |
| I am involved in research (e.g. through my own research projects or research supporting activities). | 0.259 | -0.030 | -0.085 | 0.505 | 0.118 | 0.287 |
| I seek additional learning opportunities to recognize how decisions are made in the light of new knowledge and information. | 0.063 | 0.210 | 0.100 | 0.522 | -0.087 | 0.047 |
| I support other students in their studies (e.g. as a mentor or by providing learning materials). | 0.243 | 0.030 | 0.056 | 0.314 | 0.255 | 0.084 |
| I seize learning opportunities to understand the basic principles of healthcare financing. | 0.052 | -0.041 | 0.009 | 0.734 | -0.023 | 0.037 |
| During my clinical training, I contemplate the use of resources (e.g. when ordering laboratory diagnostics). | 0.080 | 0.128 | 0.060 | 0.291 | -0.062 | -0.006 |
| I discuss the opportunities and limitations of change projects in student groups (e.g. the introduction of digital medical records). | 0.075 | 0.027 | 0.124 | 0.598 | 0.182 | 0.024 |
| When changes are introduced in medical procedures (e.g. shortening the length of inpatient treatment), I keep myself informed about their effectiveness. | 0.064 | 0.067 | 0.232 | 0.461 | -0.021 | 0.057 |
| I am involved in the student council and/or committees. | -0.265 | -0.137 | -0.053 | 0.235 | 0.516 | 0.150 |
| I take part in projects or committees to improve undergraduate medical studies and teaching. | -0.092 | 0.024 | 0.020 | 0.194 | 0.689 | 0.088 |
| I am involved in student groups to improve the general conditions for studying (e.g. support for students with children). | -0.287 | -0.003 | 0.268 | 0.095 | 0.495 | 0.067 |
| I take on leadership roles in a student group to implement teaching innovations (e.g. ultrasound tutorials). | 0.103 | -0.022 | -0.032 | 0.036 | 0.933 | 0.045 |
| I am involved in student groups to implement teaching innovations (e.g. ultrasound tutorials). | 0.015 | 0.078 | -0.067 | -0.148 | 0.985 | -0.015 |
| I take responsibility for finances or resource planning in an organization (e.g. in a club or a group). | 0.003 | -0.099 | -0.104 | 0.096 | -0.024 | 0.930 |
| I am actively involved in a change project (e.g. a reorganization in a club). | -0.093 | -0.066 | 0.158 | -0.028 | 0.232 | 0.889 |
| I share information so that others can understand me better. | 0.141 | 0.164 | 0.122 | -0.072 | 0.113 | 0.154 |
| I am able to steer group dynamic processes (e.g. by involving quieter group participants). | 0.116 | 0.080 | 0.132 | -0.073 | -0.075 | 0.304 |

Factor 1: Achieving learning and reflecting on performance; Factor 2: Demonstrating responsible behaviour and shaping relations; Factor 3: Fostering personal development and promoting quality improvement; Factor 4: Developing self-management and supporting management in healthcare; Factor 5: Promoting improvement and innovation in undergraduate medical education; Factor 6: Introducing systemic perspectives into organizations
