# Supplementary material for: Medical students‘ leadership competence in health care: development of a self-assessment scale
Source: BMC Med Educ. 2024 Nov 6;24:1275. doi: 10.1186/s12909-024-06037-2 (PMC11542227; doi:10.1186/s12909-024-06037-2)
Supplement: Supplementary file 3 — Supplement 3: MeLeCoS p-values and confidence intervals [file 12909_2024_6037_MOESM3_ESM.docx]

**Supplement 3**: MeLeCoS p-values and confidence intervals

| **Items** | **Estimate** | **CI** | **p** | **Standard error** | **Factor** |
| --- | --- | --- | --- | --- | --- |
| I question whether I have delivered the best possible performance. | 0.545 | [0.428, 0.662] | 0.000 | 0.060 |  |
| I reflect on my performance at the end of each study period or semester. | 0.463 | [0.249, 0.676] | 0.000 | 0.109 |  |
| I can control my self-learning well (e.g. I start studying early for exams). | 0.486 | [0.311, 0.661] | 0.000 | 0.089 | **1** |
| I compare my knowledge and practices with those of my peers to question both content and actions. | 0.424 | [0.260, 0.588] | 0.000 | 0.084 |  |
| I communicate goals clearly in working or learning groups so that we can work together to achieve them. | 0.337 | [0.192, 0.482] | 0.000 | 0.074 |  |
| In controversial discussions, I make sure that the views of all participants are heard before decisions are made. | 0.326 | [0.141, 0.512] | 0.001 | 0.095 |  |
| I take responsibility for the active role assigned to me in a team (e.g. minute taker). | 0.160 | [0.006, 0.315] | 0.042 | 0.079 |  |
| I motivate others in group work. | 0.187 | [0.030, 0.344] | 0.019 | 0.800 |  |
| I behave responsibly during my studies (e.g. I contribute to a good working atmosphere during group work). | 0.271 | [0.144, 0.397] | 0.000 | 0.065 |  |
| I behave ethically towards fellow students and teachers (e.g. I do not discriminate against anyone on the basis of cultural origin). | 0.334 | [0.215, 0.453] | 0.000 | 0.061 | **2** |
| I behave responsibly during clinical training, e.g. during a clinical clerkship. | 0.213 | [0.110, 0.316] | 0.000 | 0.053 |  |
| I behave ethically towards patients in clinical situations (e.g. I treat all patients equally, regardless of their social background). | 0.280 | [0.195, 0.366] | 0.000 | 0.044 |  |
| I can build a professional relationship with patients. | 0.272 | [0.163, 0.381] | 0.000 | 0.056 |  |
| In history taking, I encourage patients to share their perspective. | 0.348 | [0.206, 0.491] | 0.000 | 0.073 |  |
| I use information from others, e.g. feedback, to continue my learning. | 0.228 | [0.088, 0.368] | 0.001 | 0.071 |  |
| If I recognize the influence of poor performance on the quality of results, then I discuss this with the people involved. | 0.728 | [0.575, 0.881] | 0.000 | 0.078 |  |
| In groups, I try to discuss identified problems further. | 0.206 | [0.056, 0.357] | 0.007 | 0.077 | **3** |
| After critical incidents, I voluntarily participate in the review of work processes in the affected work area. | 0.464 | [0.266, 0.663] | 0.000 | 0.101 |  |
| I look for role models from whom I can learn something about the healthcare system or healthcare organizations. | 0.231 | [0.025, 0.437] | 0.028 | 0.105 |  |
| I organize additional extracurricular learning opportunities for myself (e.g. study groups with fellow students). | 0.256 | [0.042, 0.470] | 0.019 | 0.109 |  |
| In emotional situations, e.g. when receiving very critical feedback, I communicate in a controlled and objective manner. | 0.298 | [0.156, 0.441] | 0.000 | 0.073 |  |
| I am involved in research (e.g. through my own research projects or research supporting activities). | 0.505 | [0.243, 0.767] | 0.000 | 0.134 |  |
| I seek additional learning opportunities to recognize how decisions are made in the light of new knowledge and information. | 0.522 | [0.310, 0.735] | 0.000 | 0.108 |  |
| I support other students in their studies (e.g. as a mentor or by providing learning materials). | 0.314 | [0.084, 0.544] | 0.008 | 0.117 | **4** |
| I seize learning opportunities to understand the basic principles of healthcare financing. | 0.734 | [0.544, 0.923] | 0.000 | 0.097 |  |
| During my clinical training, I contemplate the use of resources (e.g. when ordering laboratory diagnostics). | 0.291 | [0.139, 0.443] | 0.000 | 0.077 |  |
| I discuss the opportunities and limitations of change projects in student groups (e.g. the introduction of digital medical records). | 0.598 | [0.374, 0.822] | 0.000 | 0.114 |  |
| When changes are introduced in medical procedures (e.g. shortening the length of inpatient treatment), I keep myself informed about their effectiveness. | 0.461 | [0.279, 0.643] | 0.000 | 0.093 |  |
| I am involved in the student council and/or committees. | 0.516 | [0.315, 0.716] | 0.000 | 0.102 |  |
| I take part in projects or committees to improve undergraduate medical studies and teaching. | 0.689 | [0.487, 0.890] | 0.000 | 0.103 |  |
| I am involved in student groups to improve the general conditions for studying (e.g. support for students with children). | 0.495 | [0.280, 0.710] | 0.000 | 0.110 | **5** |
| I take on leadership roles in a student group to implement teaching innovations (e.g. ultrasound tutorials). | 0.933 | [0.734, 1.133] | 0.000 | 0.102 |  |
| I am involved in student groups to implement teaching innovations (e.g. ultrasound tutorials). | 0.985 | [0.792, 1.179] | 0.000 | 0.990 |  |
| I take responsibility for finances or resource planning in an organization (e.g. in a club or a group). | 0.930 | [0.663, 1.197] | 0.000 | 0.136 |  |
| I am actively involved in a change project (e.g. a reorganization in a club). | 0.889 | [0.636, 1.143] | 0.000 | 0.129 | **6** |
| I share information so that others can understand me better. | 0.154 | [0.027, 0.282] | 0.017 | 0.065 |  |
| I am able to steer group dynamic processes (e.g. by involving quieter group participants). | 0.304 | [0.137, 0.472] | 0.000 | 0.085 |  |

Factor 1: Achieving learning and reflecting on performance; Factor 2: Demonstrating responsible behaviour and shaping relations; Factor 3: Fostering personal development and promoting quality improvement; Factor 4: Developing self-management and supporting management in healthcare; Factor 5: Promoting improvement and innovation in undergraduate medical education; Factor 6: Introducing systemic perspectives into organizations
