# Supplementary material for: Medical students‘ leadership competence in health care: development of a self-assessment scale
Source: BMC Med Educ. 2024 Nov 6;24:1275. doi: 10.1186/s12909-024-06037-2 (PMC11542227; doi:10.1186/s12909-024-06037-2)
Supplement: Supplementary file 4 — Supplement 4: MeLeCoS item scale correlations [file 12909_2024_6037_MOESM4_ESM.docx]

**Supplement 4**: MeLeCoS item-scale correlations

| **Items** | **Item-scale correlation** | **Factor**  **No.** |
| --- | --- | --- |
| I question whether I have delivered the best possible performance. | 0.593 | **1** |
| I reflect on my performance at the end of each study period or semester. | 0.518 |  |
| I can control my self-learning well (e.g. I start studying early for exams). | 0.500 |  |
| I compare my knowledge and practices with those of my peers to question both content and actions. | 0.568 |  |
| I communicate goals clearly in working or learning groups so that we can work together to achieve them. | 0.444 |  |
| In controversial discussions, I make sure that the views of all participants are heard before decisions are made. | 0.459 | **2** |
| I take responsibility for the active role assigned to me in a team (e.g. minute taker). | 0.335 |  |
| I motivate others in group work. | 0.441 |  |
| I behave responsibly during my studies (e.g. I contribute to a good working atmosphere during group work). | 0.444 |  |
| I behave ethically towards fellow students and teachers (e.g. I do not discriminate against anyone on the basis of cultural origin). | 0.392 |  |
| I behave responsibly during clinical training, e.g. during a clinical clerkship. | 0.417 |  |
| I behave ethically towards patients in clinical situations (e.g. I treat all patients equally, regardless of their social background). | 0.414 |  |
| I can build a professional relationship with patients. | 0.495 |  |
| In history taking, I encourage patients to share their perspective. | 0.413 |  |
| I use information from others, e.g. feedback, to continue my learning. | 0.493 | **3** |
| If I recognize the influence of poor performance on the quality of results, then I discuss this with the people involved. | 0.568 |  |
| In groups, I try to discuss identified problems further. | 0.428 |  |
| After critical incidents, I voluntarily participate in the review of work processes in the affected work area. | 0.456 |  |
| I look for role models from whom I can learn something about the healthcare system or healthcare organizations. | 0.391 |  |
| I organize additional extracurricular learning opportunities for myself (e.g. study groups with fellow students). | 0.369 |  |
| In emotional situations, e.g. when receiving very critical feedback, I communicate in a controlled and objective manner. | 0.403 | **4** |
| I am involved in research (e.g. through my own research projects or research supporting activities). | 0.456 |  |
| I seek additional learning opportunities to recognize how decisions are made in the light of new knowledge and information. | 0.502 |  |
| I support other students in their studies (e.g. as a mentor or by providing learning materials). | 0.360 |  |
| I seize learning opportunities to understand the basic principles of healthcare financing. | 0.585 |  |
| During my clinical training, I contemplate the use of resources (e.g. when ordering laboratory diagnostics). | 0.409 |  |
| I discuss the opportunities and limitations of change projects in student groups (e.g. the introduction of digital medical records). | 0.562 |  |
| When changes are introduced in medical procedures (e.g. shortening the length of inpatient treatment), I keep myself informed about their effectiveness. | 0.559 |  |
| I am involved in the student council and/or committees. | 0.595 | **5** |
| I take part in projects or committees to improve undergraduate medical studies and teaching. | 0.616 |  |
| I am involved in student groups to improve the general conditions for studying (e.g. support for students with children). | 0.518 |  |
| I take on leadership roles in a student group to implement teaching innovations (e.g. ultrasound tutorials). | 0.664 |  |
| I am involved in student groups to implement teaching innovations (e.g. ultrasound tutorials). | 0.643 |  |
| I take responsibility for finances or resource planning in an organization (e.g. in a club or a group). | 0.506 | **6** |
| I am actively involved in a change project (e.g. a reorganization in a club). | 0.576 |  |
| I share information so that others can understand me better. | 0.353 |  |
| I am able to steer group dynamic processes (e.g. by involving quieter group participants). | 0.381 |  |

Factor 1: Achieving learning and reflecting on performance; Factor 2: Demonstrating responsible behaviour and shaping relations; Factor 3: Fostering personal development and promoting quality improvement; Factor 4: Developing self-management and supporting management in healthcare; Factor 5: Promoting improvement and innovation in undergraduate medical education; Factor 6: Introducing systemic perspectives into organizations
